# Similarity of Shiga Toxin–producing *Escherichia coli* O104:H4 Strains from Italy and Germany

**DOI:** 10.3201/eid1710.111072

**Published:** 2011-10

**Authors:** Gaia Scavia, Stefano Morabito, Rosangela Tozzoli, Valeria Michelacci, Maria Luisa Marziano, Fabio Minelli, Clarissa Ferreri, Fabio Paglialonga, Alberto Edefonti, Alfredo Caprioli

**Affiliations:** Istituto Superiore di Sanità, Rome, Italy (G. Scavia, S. Morabito, R. Tozzoli, V. Michelacci, M.L. Marziano, F. Minelli, C. Ferreri, A. Caprioli);; Ospedale Maggiore Policlinico, Milan, Italy (F. Paglialonga, A. Edefonti)

**Keywords:** enteric infections, Escherichia coli, Shiga toxin, hemolytic uremic syndrome, bacteria, Italy, Germany, outbreak, strains, foodborne infections, letter

**To the Editor:** Since the beginning of May 2011, a large outbreak of infections associated with Shiga toxin (Stx)–producing *Escherichia coli* (STEC) O104:H4 has occurred in Germany (*1*). The outbreak showed 3 unusual features: 1) a large proportion of case-patients with hemolytic uremic syndrome (HUS); 2) HUS in adults, although it usually affects children; and 3) frequent development of neurologic symptoms in patients when clinical and laboratory markers of HUS were improving (*1**,**2*). A second point-source outbreak caused by the same STEC O104 strain was reported in June 2011 in France (*3*). Both outbreaks were linked to eating fenugreek sprouts obtained from seeds produced in Egypt and distributed in Germany and other European countries (*4*).

Instead of the attaching–effacing mechanism of adhesion to intestinal mucosa that is typical of STEC associated with severe human disease (*5*), the STEC O104 epidemic strain had genetic markers and an adhesion pattern (*6*) typical of enteroaggregative *E. coli* (EAEC), another group of diarrheagenic strains found frequently in developing countries (*5*).

On basis of these findings, we reviewed our culture collection and found that an STEC strain (ED-703) from a case-patient with HUS in 2009 in Italy had the same combination of virulence factors as the strain from Germany: Stx2 production and enteroaggregative adhesion genetic markers. This strain, which had not been typed when it was isolated, showed positive PCR results for O104 (*7*) and H4 (*8*) antigen–associated genes and was agglutinated by an O104 antiserum (Statens Serum Institut, Copenhagen, Denmark). Pulsed-field gel electrophoresis showed a high degree of similarity (94.7%) with the outbreak strain from Germany (provided by M. Mielke, Robert Koch Institute, Berlin, Germany). In contrast with the outbreak strain, ED-703 did not produce extended-spectrum β-lactamases.

The strain from our culture collection had been isolated from a 9-year-old girl admitted to the pediatric nephrology unit of the Ospedale Maggiore (Milan, Italy) on August 5, 2009, after 5 days of bloody diarrhea, vomiting, and abdominal pain. Diagnosis of HUS was based on the presence of hemolytic anemia, thrombocytopenia, and anuria. Neurologic symptoms (e.g., lethargy, diplopia, and nystagmus) occurred during hospitalization; magnetic resonance imaging showed signal abnormalities in the lenticular nuclei.

Because of severe cardiac impairment with ejection fraction reduction and troponin increase, inotropic support and mechanical ventilation were temporarily needed. After improvement of clinical conditions, the patient was discharged, but she was readmitted a few days later because of headache, vomiting, confusion, dysarthria, hypertension, and visual impairment. Ischemic lesions were found by magnetic resonance imaging at fundus oculi. Neurologic status improved the next day, but the visual deficit persisted. Hemodialysis was needed for 2 months. Long-term sequelae of the disease were stage IV chronic kidney disease, hypertension, and severe visual impairment.

Informed consent and an epidemiologic interview were obtained from the patient’s parents. The household, including her mother and 2 siblings (4 and 5 years of age), had traveled for 1 week to a resort in Tunisia; they had returned 3 weeks before the onset of the prodromal symptoms of HUS. Four days after their return, the youngest sister was hospitalized for 3 days because of bloody diarrhea, but no laboratory diagnosis was established. The mother reported having had watery diarrhea and abdominal pain on August 2. The patient history did not show any other usual risk factor for STEC infection, such as consumption of unpasteurized milk or dairy products, undercooked meat, or raw sprouts or direct exposure to ruminants or their manure. This finding suggests that the infection was probably acquired through person-to-person transmission.

This case report confirms that strains of STEC O104 strictly related to the epidemic strain in Germany had already caused sporadic infections in Europe (*9*). Other cases have been documented in 2001 in Germany (*6**,**9*), in 2004 in France (*9*), and in 2010 in Finland in a patient with diarrhea who had traveled to Egypt (*9*). Both of the cases for which the information on the origin of the infection was available were related to travel to northern Africa, from which the seeds associated with both outbreaks could be traced (*4*).

The history of this patient supports the hypothesis that ruminants would not have had a specific role in the transmission of STEC O104:H4, as already suggested by the epidemiologic features of the recent outbreaks (*1**,**3*). In fact, STEC O104 cannot be considered true STEC but rather EAEC strains that acquired the Stx2-coding phages by horizontal gene transfer, and EAEC is considered to be a human pathogen usually transmitted by the oral–fecal route (*5*).

The clinical course of our patient closely resembles those of persons who had HUS associated with the German outbreak (*1**,**2*). The unusual combination of virulence factors of STEC and EAEC, already described in a group of STEC O111:H2 from an outbreak of HUS in France in 1996 (*10*), might confer a high degree of virulence to these strains. It also might explain the severity of the clinical findings associated with STEC O104:H4 infections.
